# Ablation Via the Left Atrial Appendage

**DOI:** 10.1186/1749-8090-10-S1-A45

**Published:** 2015-12-16

**Authors:** David H Savage

**Affiliations:** 1 Department of Surgery, Piedmont Medical Center, Rock Hill, SC, 29732, USA

## Background/Introduction

Despite its proven efficacy, the left atrial lesion set of the Cox-maze III operation is not universally employed during coronary artery bypass or aortic valve surgery for patients with atrial fibrillation, in part due to the surgeon's reluctance to add bicaval cannulation and a left atriotomy not needed for the primary procedure.

## Aims/Objectives

To describe a less invasive technique to create a complete lesion set encircling all pulmonary veins and a connecting lesion to the mitral annulus that does not require bicaval cannulation or an atriotomy in addition to amputation of the left atrial appendage.

## Method

Fourteen consecutive patients with long term and short term persistent atrial fibrillation were the basis of this report. On full cardiopulmonary bypass, radiofrequency clamps were applied to the epicardial left atrial tissue surrounding the right and left pulmonary veins, separately. The left atrial appendage was amputated on the arrested heart. Via that defect, an epicardial to endocardial radiofrequency ablation lesion was created with the clamp to the left superior pulmonary vein, then to the right superior pulmonary vein. A cryothermy catheter was then placed to create an endocardial lesion from the left inferior pulmonary vein ablation mark, along the posterior left atrial floor, to the right inferior pulmonary vein. The catheter then connected that floor lesion to the mitral valve annulus at P2-P3. Right atrial lesions were performed in 8 patients in the manner described for the Cox-maze IV operation.

## Results

By EKG and symptoms, all 14 patients were free of atrial fibrillation at a mean follow up of 11 months (range 3-17months). There was no mortality in this cohort either at operation or during follow up and no complications from left atrial access.

## Discussion/Conclusion

The amputated left atrial appendage stump can be used as a window to create a full pulmonary vein box lesion set and connecting lesion to the annulus of the mitral valve. This simplified technique could allow more wide spread use of the left atrial Cox-maze III lesion set in concomitant atrial fibrillation ablation procedures. Further study will be needed to assess the durability of this approach.

